# Long-lasting subjective effects of LSD in normal subjects

**DOI:** 10.1007/s00213-017-4733-3

**Published:** 2017-09-16

**Authors:** Yasmin Schmid, Matthias E. Liechti

**Affiliations:** Division of Clinical Pharmacology and Toxicology, Department of Biomedicine, Department of Clinical Research, University Hospital Basel, University of Basel, Schanzenstrasse 55, 4056 Basel, Switzerland

**Keywords:** LSD, Mystical experience, Personality, Hallucinogen, Humans

## Abstract

**Rationale:**

Lysergic acid diethylamide (LSD) and other serotonergic hallucinogens can induce profound alterations of consciousness and mystical-type experiences, with reportedly long-lasting effects on subjective well-being and personality.

**Methods:**

We investigated the lasting effects of a single dose of LSD (200 μg) that was administered in a laboratory setting in 16 healthy participants. The following outcome measures were assessed before and 1 and 12 months after LSD administration: Persisting Effects Questionnaire (PEQ), Mysticism Scale (MS), Death Transcendence Scale (DTS), NEO-Five Factor Inventory (NEO-FFI), and State-Trait Anxiety Inventory (STAI).

**Results:**

On the PEQ, positive attitudes about life and/or self, positive mood changes, altruistic/positive social effects, positive behavioral changes, and well-being/life satisfaction significantly increased at 1 and 12 months and were subjectively attributed by the subjects to the LSD experience. Five-Dimensions of Altered States of Consciousness (5D-ASC) total scores, reflecting acutely induced alterations in consciousness, and Mystical Experience Questionnaire (MEQ30) total scores correlated with changes in well-being/life satisfaction 12 months after LSD administration. No changes in negative attitudes, negative mood, antisocial/negative social effects, or negative behavior were attributed to the LSD experience. After 12 months, 10 of 14 participants rated their LSD experience as among the top 10 most meaningful experiences in their lives. Five participants rated the LSD experience among the five most spiritually meaningful experiences in their lives. On the MS and DTS, ratings of mystical experiences significantly increased 1 and 12 months after LSD administration compared with the pre-LSD screening. No relevant changes in personality measures were found.

**Conclusions:**

In healthy research subjects, the administration of a single dose of LSD (200 μg) in a safe setting was subjectively considered a personally meaningful experience that had long-lasting subjective positive effects.

**Trial registration:**

Registration identification number: NCT01878942.

**Electronic supplementary material:**

The online version of this article (10.1007/s00213-017-4733-3) contains supplementary material, which is available to authorized users.

## Introduction

Classic hallucinogens, including d-lysergic acid diethylamide (LSD), psilocybin, and dimethyltryptamine (which is contained in ayahuasca), produce their psychotropic effects by acting on serotonin 5-hydroxytryptamine-2A (5-HT_2A_) receptors (Preller et al. 2017; Rickli et al. 2016). These serotonergic hallucinogens are used for recreational, personal/spiritual, and therapeutic purposes (Nichols 2016). Approximately 10% of US residents reported having used LSD at least once (Johnston et al. 2016; Krebs and Johansen 2013).

The use of LSD as an adjunct to psychotherapy has been associated with positive health outcomes in patients with anxiety that was caused by life-threatening diseases (Gasser et al. 2014, 2015; Pahnke et al. 1970), depression (Rucker et al. 2016), and substance use disorder (Krebs and Johansen 2012; Savage and McCabe 1973). Additionally, self-reports indicated positive lasting effects on values, attitudes, personality, and well-being that were attributed to LSD administration (200 μg p.o.) in 24 psychiatrically healthy subjects (McGlothlin et al. 1967). However, more recent data on similar long-term effects of experimentally administered LSD in normal subjects are lacking.

Clinical research on LSD has only very recently been restarted after no clinical studies were conducted for 40 years (Liechti 2017). With the exception of one clinical therapeutic trial (Gasser et al. 2014), all recent research on LSD had an experimental focus on aspects of the acute effects of LSD in healthy participants (Carhart-Harris et al. 2016a, b; Dolder et al. 2016; Liechti 2017; Liechti et al. 2017; Mueller et al. 2017; Preller et al. 2017; Schmid et al. 2015). Only one recent study investigated lasting effects and reported increases in trait optimism and openness 2 weeks after LSD (75 μg i.v.) administration in 20 healthy subjects (Carhart-Harris et al. 2016a). In contrast to LSD, considerably more recent research has been conducted on psilocybin using doses up to 0.43 mg/kg p.o., including reports of positive long-term effects in a total of 129 healthy subjects (Griffiths et al. 2006, 2008, 2011, 2017; MacLean et al. 2011) and a total of 105 patients (Bogenschutz et al. 2015; Griffiths et al. 2016; Johnson et al. 2016; Ross et al. 2016). Psilocybin has been shown to induce persisting positive changes in attitudes, social effects, mood, life satisfaction, behavior, and trait openness in healthy volunteers up to 14 months after administration (Griffiths et al. 2006, 2008, 2011, 2017; MacLean et al. 2011). Lasting positive effects of hallucinogens have been linked to their ability to acutely induce profound insights and mystical-type experiences (Pahnke 1969). Such acute mystical-type effects of psilocybin have now been characterized in more recent studies including also validated scales such as the Mystical Experience Questionnaire (MEQ; Barrett et al. 2015) and Mysticism Scale (MS; Hood et al. 2001; Hood 1975) in healthy participants (Barrett and Griffiths 2017; Barrett et al. 2015; Garcia-Romeu et al. 2015; Griffiths et al. 2006, 2008, 2011, 2017; MacLean et al. 2011). Additionally, these effects have been correlated with lasting changes after psilocybin administration (Barrett et al. 2015; Griffiths et al. 2008, 2017; MacLean et al. 2011).

The goal of the present study was to describe the persisting effects of LSD 1 and 12 months after administration of a single dose (200 μg p.o.) in 16 healthy participants using psychometric instruments that were not validated in German but that were similar to those used in studies of psilocybin (Griffiths et al. 2006, 2008, 2011, 2017). Based on previous research on psilocybin, the first a priori study hypothesis was that the LSD experience would be self-rated by the participants on the Persisting Effects Questionnaire (PEQ) as personally meaningful, spiritually significant, and positively influencing their sense of well-being up to 12 months, similar to reports for psilocybin (Griffiths et al. 2006, 2008, 2011, 2017). We also hypothesized that LSD would increase total ratings of mystical experiences on the Mysticism Scale (MS; lifetime version) and total ratings on the Death Transcendence Scale (DTS) 1 and 12 months after administration compared with pre-LSD screening (Griffiths et al. 2006, 2011). We further expected that the acute alterations in consciousness (Five Dimensions of Altered States of Consciousness [5D-ASC] total score) and mystical-type experiences (MEQ30 total score and MS total score) that are induced by LSD would correlate with its persisting effects (PEQ, MS total score, and DTS total score; Griffiths et al. 2008; Griffiths et al. 2017). Finally, we hypothesized that LSD would produce lasting increases in personality trait openness, similar to the effects of psilocybin (MacLean et al. 2011).

## Methods

### Study design

The present study included a priori-planned short-term (1 month) and long-term (12 months) follow-ups after the administration of a single dose of LSD and placebo using a randomized, double-blind, cross-over study with two experimental test sessions in balanced order. The washout periods between study sessions were at least 7 days. The study was conducted in accordance with the Declaration of Helsinki and International Conference on Harmonization Guidelines in Good Clinical Practice (ICH-GCP) and approved by the Ethics Committee of the Canton of Basel, Switzerland, and the Swiss Agency for Therapeutic Products (Swissmedic). The administration of LSD in healthy subjects was authorized by the Swiss Federal Office for Public Health, Bern. The study was registered at ClinicalTrials.gov (NCT01878942). All of the subjects provided written informed consent and were paid for their participation. The present clinical study also assessed additional acute subjective, autonomic, adverse, endocrine, and pharmacokinetic effects of LSD as reported in detail elsewhere (Dolder et al. 2015, 2016, 2017; Liechti et al. 2017; Schmid et al. 2015; Strajhar et al. 2016). In the present paper, we report the short- and long-term follow-up safety data and the sustained effects of LSD.

### Participants

Sixteen healthy subjects (eight men and eight women; mean age ± SD 28.6 ± 6.2 years; range 25–51 years) were recruited by word of mouth or an advertisement that was placed on the web market platform of the University of Basel. All of the subjects were well-educated. Ten participants had at least a masters degree, and four were graduate students. The exclusion criteria were age < 25 years or > 65 years, pregnancy, personal or first-degree relative history of psychotic or major affective disorder, chronic or acute illness, and illicit drug use within the last 2 months or during the study period. Seven subjects had used a hallucinogen one to three times. Nine subjects were hallucinogen-naive. The exclusion criteria and drug use histories are reported in detail elsewhere (Schmid et al. 2015). Additionally, we considered the safety recommendations for high-dose hallucinogen research (Johnson et al. 2008).

### Study procedures

The study included a 1–2 h screening visit with the study physician, a separate 1 h psychiatric interview, two 25 h test sessions, a 1-h follow-up visit approximately 1 month (mean ± SD 37 ± 14 days) after the LSD test session, and a follow-up mail contact that was scheduled at least 12 months (491 ± 199 days) after LSD administration. The two test sessions included the administration of either LSD or placebo. The sessions lasted 25 h each and were conducted in a calm and neutral hospital research ward environment. The test sessions began at 8:15 a.m. LSD (200 μg﻿) or placebo was administered at 9:00 a.m. The subjects were under constant supervision by the study physician until the drug effects ceased up to 16 h after drug administration (1:00 a.m.). Thus, the subjects were never alone during the first 16 h after drug administration, and the investigator was in a room next to the subject during the night up to 24 h. The participants were resting in hospital beds. They could interact with the investigator, rest, or listen to music via headphones up to 1:00 a.m., but no further entertainment was provided and the night was spent sleeping (1:00 a.m.–08:00 a.m.). The first follow-up was scheduled approximately 1 month after the LSD session as a hospital visit. The participants completed questionnaires that assessed lasting effects (see the “Outcome measures” section below) and were asked about adverse events, and a physical examination was performed. The participants were asked to report any psychological problems, including depressed mood, sleeping problems, somatic disorders, and any perceptual changes/disorders (e.g., flashbacks). The subjects were contacted again by regular mail or/and e-mail and asked to complete the same questionnaires at least 12 months after the LSD session.

### Drugs

LSD was administered in a single oral dose of 200 μg (d-lysergic acid diethylamide base; Lipomed AG, Arlesheim, Switzerland). The same dose and gelatin capsule formulation was used in LSD-assisted psychotherapy in a clinical study (Gasser et al. 2014). The dose was within the range of doses that are taken for recreational purposes and expected to induce robust effects in humans (Passie et al. 2008).

### Outcome measures

The present LSD study used the same acute and long-term outcome measures as the previous psilocybin studies, including the PEQ, MS, DTS, and similar and additional personality measures (Griffiths et al. 2006, 2008, 2011; MacLean et al. 2011). The PEQ, MS, and DTS were unavailable in validated German versions. Therefore, the original English questionnaires (Griffiths et al. 2006, 2008, 2011) were independently forward-translated into German by two translators with German as their mother tongue. Discrepancies between the two forward-translated versions and other previous German versions were then discussed, and selected items were backtranslated. The versions were then pretested for comprehension by people with prior LSD use. The non-validated German versions that were finally used in the study are available online (Supplementary Appendices 1–3).

#### Persisting Effects Questionnaire

The 143-item PEQ is a non-validated questionnaire that has previously been used to study long-term effects of psilocybin (Griffiths et al. 2006, 2008, 2011). A non-validated German version was used (Supplementary Appendix 1). A total of 140 items are rated on six-point Likert scales to assess possible changes in positive or negative attitudes about life and/or self (17 items each), positive and negative mood changes (4 items each), altruistic/positive and antisocial/negative social effects (8 items each), and positive and negative behavioral changes (1 item each; Griffiths et al. 2006). All ratings reflect persisting changes that are subjectively related to the LSD experience, and no change would be reflected by a score of 0. Three additional questions were included as previously described (Griffiths et al. 2006, 2011): (1) How personally meaningful was the experience? (1 = no more than routine, everyday experiences; 2 = similar to meaningful experiences that occur on average once or more per week; 3 = similar to meaningful experiences that occur on average once per month; 4 = similar to meaningful experiences that occur on average once per year; 5 = similar to meaningful experiences that occur on average once every 5 years; 6 = among the 10 most meaningful experiences of my life; 7 = among the 5 most meaningful experiences of my life; 8 = the single most meaningful experience of my life); (2) indicate the degree to which the experience was spiritually significant for you (1 = not at all; 2 = slightly; 3 = moderately; 4 = very much; 5 = among the 5 most spiritually significant experiences of my life; 6 = the single most spiritually significant experience of my life); and (3) do you believe that the experience and your contemplation of the experience have led to a change in your current sense of personal well-being or life satisfaction? (+ 3 = increased very much to − 3 = decreased very much; 0 indicates no change; Griffiths et al. 2006, 2008, 2011). The questionnaires were completed 1 and 12 months after LSD administration. One subject was lost to follow-up at 12 months, one subject did not complete all of the questions at 1 and 12 months, and one subject did not complete all of the questions at 1 month, thus resulting in data from 14 subjects at both time points.

#### Mysticism Scale

The MS is a validated 32-item questionnaire that was developed to assess primary mystical experiences (Hood et al. 2001; Hood 1975). The MS has previously been used to measure mystical experiences that were induced by psilocybin (Griffiths et al. 2006, 2008, 2011; MacLean et al. 2011). A non-validated German version was used (Supplementary Appendix 2). The items were rated on a nine-point Likert scale (− 4 = this description is extremely not true of my own experience or experiences to + 4 = this description is extremely true of my own experience or experiences). The scale consists of 16 positively worded statements and 16 negatively worded statements. Negative items were reverse scored and summed with the positive items. A total score and three empirically derived factors were calculated as previously described: interpretation (corresponding to noetic quality, sacredness, and deeply felt positive mood), introvertive mysticism (corresponding to internal unity, transcendence of time and space, and ineffability), and extrovertive mysticism (corresponding to unity of all things/all things are alive; Griffiths et al. 2006). The MS was completed 24 h after the administration of LSD and placebo, and the participants were asked to retrospectively rate their experiences relative to their experiences since receiving the drug that morning. For the lifetime version, the participants were instructed to answer the questions relative to their total life experiences. The lifetime version was used at screening and 1 and 12 months after LSD administration. One subject was lost to follow-up at 12 months, and one subject did not complete all of the questions at 12 months, thus resulting in complete datasets for 14 subjects.

#### Death Transcendence Scale

The validated 26-item DTS includes five factors/subscales (mysticism, religion, nature, creativity, and biosocial) reflecting different aspects of death transcendence (Hood and Morris 1983; VandeCreek 1999; VandeCreek and Nye 1993). A non-validated German version (Supplementary Appendix 3) was administered at screening and 1 and 12 months after LSD administration. The items were rated on a sevent-point scale. One subject was lost to follow-up at 12 months, and one subject did not complete all of the questions at 12 months, thus resulting in 14 complete datasets.

#### Personality measures

The 60-item NEO-Five-Factor Inventory (NEO-FFI; Borkenau and Ostendorf 2008) was derived from the NEO Personality Inventory (Costa and McCrae 1995) and used to assess personality traits. The Spielberger State-Trait Anxiety Inventory (STAI; Form X2) was used to assess trait anxiety (Spielberger et al. 1970). Measures were taken at screening and 1 and 12 months after LSD administration. One subject was lost to follow-up at 12 months, thus resulting in 15 complete datasets.

#### Additional measures of acute mystical-type and mind-altering effects of LSD

Two of the previously described measures of the acute mystical-type and mind-altering effects of LSD (Liechti et al. 2017) were again used in the present data analysis to assess whether the acute response to LSD on these scales predicted its long-term effects in the follow-up questionnaires as similarly evaluated for psilocybin in healthy subjects and using the same psychometric scales (Griffiths et al. 2008, 2011, 2017; MacLean et al. 2011). These two measures were the MEQ (Barrett et al. 2015; Griffiths et al. 2006; MacLean et al. 2012) and the 5D-ASC (Studerus et al. 2010). A German version (Liechti et al. 2017) of the 43-item MEQ (Griffiths et al. 2006; MacLean et al. 2012; Pahnke 1969) was used, including six subscales (internal unity, external unity, sacredness, noetic quality, deeply felt positive mood, transcendence of time/space, and ineffability). We also derived the four scale scores of the newly validated revised 30-item MEQ (mystical, positive mood, transcendence of time and space, and ineffability; Barrett et al. 2015). The 5D-ASC was used to assess the peak alterations of consciousness. The 5D-ASC contains 94 items (visual analog scales; Dittrich 1998; Studerus et al. 2010). The instrument consists of five subscales/dimensions (Dittrich 1998). The 5D-ASC dimension “Oceanic Boundlessness” (OB, 27 items) measures derealization and depersonalization associated with positive emotional states, ranging from heightened mood to euphoric exaltation. The dimension “Anxious Ego Dissolution” (AED, 21 items) summarizes ego-disintegration and loss of self-control phenomena associated with anxiety. The dimension “Visionary Restructuralization” (VR, 18 items) describes perceptual changes including complex imagery, elementary imagery, audio-visual synesthesia, and changed meaning of percepts. Two additional dimensions describe “Auditory Alterations” (AA, 15 items) and “Reduction of Vigilance” (12 items). The total 5D-ASC score is the total of all dimensions.

The MEQ and 5D-ASC were administered 24 h after drug administration, and the participants were asked to retrospectively rate drug effects during peak drug effects. Greater acute effects of psilocybin on the MEQ43 and 5D-ASC predicted lasting changes in openness in a previous study (MacLean et al. 2011), and greater acute MEQ30 ratings also predicted greater changes in well-being/life satisfaction at 6 months in another study (Griffiths et al. 2017).

### Statistical analysis

The data were analyzed using Statistica 12 (StatSoft, Tulsa, OK, USA). Paired *t* tests (two-tailed) were used to test for differences between the acute effects of LSD and placebo on the MS. Repeated-measures analysis of variance (ANOVA), with time (screening, 1 month after LSD, and 12 months after LSD) as the within-subject factor, followed by the Tukey post hoc test was used to analyze differences between screening and the two follow-up assessments over time. Sex differences were assessed by adding sex as a between-subjects factor to the ANOVA. Persisting effect ratings on the PEQ at 1 or 12 months were compared with 0 (i.e., no change compared with everyday life) using one-sample *t* tests because the PEQ ratings would be attributed to the LSD experience compared with no change, and there was no measurement at screening. Associations between the acute response to LSD and main 12-month outcome measures were assessed using Spearman rank order correlations. The criterion for significance was *P* < 0.05. The values are shown without correction for multiple comparisons because of the use of defined a priori hypotheses for most of the study outcomes.

## Results

### Acute effects of LSD on the Mysticism Scale

LSD increased the total score and all three factors of the MS compared with placebo. The total score (mean ± SEM) was 194 ± 14 and 45 ± 9 for LSD and placebo, respectively (*t*_13_ = 8.9, *P* < 0.001). Ratings of “interpretation” were 72 ± 6 and 17 ± 3 after the administration of LSD and placebo, respectively (*t*_13_ = 7.6, *P* < 0.001). Ratings of “introvertive” were 55 ± 3 and 12 ± 2 after LSD and placebo administration, respectively (*t*_13_ = 13.2, *P* < 0.001). Ratings of “extrovertive” were 67 ± 7 and 16 ± 3 after LSD and placebo administration, respectively (*t*_13_ = 7.0, *P* < 0.001). The acute effects of LSD and placebo on the 5D-ASC and MEQ have previously been reported and are summarized in Table S1 (Liechti et al. 2017). Acute administration of LSD significantly increased the total 5D-ASC and MEQ30 scores and ratings on all subscales except anxiety on the 5D-ASC (Table S1).

### Lasting effects of LSD

#### Persisting Effects Questionnaire

Ratings of positive attitudes about life and/or self, positive mood changes, altruistic/positive social effects, and positive behavioral changes significantly increased at the 1- and 12-month follow-ups compared with the assumption of no change (rating = 0; Table 1). Ratings of negative attitudes about life and/or self, negative mood changes, antisocial/negative social effects, or negative behavior changes attributed to the LSD experience (Table 1) were not different at the 1 and 12 month follow-ups compared with the assumption of no change (rating 0; Table 1). Ratings of meaningfulness of the LSD experience (“How personally meaningful was the LSD experience”) significantly increased at 1 and 12 months compared with the assumption that the experience would be as meaningful as an everyday experience (rating = 1; Table 1). After 12 months, 10 of 14 participants rated the LSD experience among the top 10 most meaningful experiences in their lives (rating ≥ 6). Among these 10 participants, five rated it among the top five most meaningful experiences (rating = 7). Ratings of spiritual significance (“Indicate the degree to which the experience was spiritually significant to you”) increased at both 1 and 12 months compared with the assumption that the experience would have no spiritual meaning (rating = 1; Table 1). At 12 months, eight of 14 participants rated the experience as very strongly spiritual (rating ≥ 4). Among these eight participants, five reported that the LSD experience was among the five most spiritually meaningful experiences in their lives (rating ≥ 5). One of these participants considered the LSD experience the most spiritually significant experience in his life (rating = 6). Ratings of well-being or life satisfaction also significantly increased compared with the assumption of no change (rating = 0; Table 1). No significant differences in these three ratings were found between the 1- and 12-month follow-ups. No subject rated the LSD experience as decreasing well-being or life satisfaction.

**Table 1:** Participant ratings (mean [SEM]) and statistics

	Screening	1 month			12 months				
Persisting Effects Questionnaire^a^			*T*_13_	*P=*		*T*_13_	*P=*		
Positive attitudes about life and/or self		35.1 (5.9)	6.23	***	39.3 (6.5)	6.24	***		
Positive mood changes		21.8 (5.0)	4.52	***	32.5 (8.2)	4.11	**		
Altruistic/positive social effects		23.2 (5.9)	4.11	**	28.4 (6.3)	4.72	***		
Positive behavior changes		30.0 (7.1)	4.36	***	30.0 (7.8)	4	**		
Negative attitudes about life and/or self		1.1 (0.6)	1.82	NS	0.5 (0.3)	2.12	NS		
Negative mood changes		1.1 (1.1)	1	NS	0.7 (0.7)	1	NS		
Antisocial/negative social effects		1.0 (0.9)	1.63	NS	1.8 (1.0)	1.85	NS		
Negative behavior changes		0.0 (0.0)	1	NS	0.0 (0.0)	1	NS		
			*T*_13_	*P=*		*T*_13_	*P=*		
How personally meaningful was the experience? (score range 1–8)^b^		5.4 (0.3)	14.17	***	5.7 (0.4)	11.8	***		
How spiritually significant was the experience? (score range 1–6)^b^		2.9 (0.5)	4.28	***	3.4 (0.5)	4.84	***		
Did the experience change your sense of well-being or life satisfaction? (score range − 3 to + 3, 0 = no change)^b^		1.0 (0.3)	3.8	**	1.4 (0.3)	4.76	***		
Mysticism Scale^b^								*F*_2*,*26_	*P=*
Interpretation (max score = 108)	65.1 (3.8)	75.9 (5.1)			76.6 (5.5)			3.32	NS
Introvertive (max score = 108)	37.9 (3.7)	52.1 (3.6)	**		52.0 (3.9)	**		8.13	< 0.01
Extrovertive (max score = 172)	47.4 (5.4)	69.5 (8.0)	**		74.4 (6.7)	***		10.43	< 0.001
Total (max score = 288)	151 (10.9)	198 (14.3)	**		203 (14.6)	***		11.13	< 0.001
Death Transcendence Scale^b^								*F*_2*,*26_	*P=*
Mysticism (max score = 35)	13.1 (2.0)	21.1 (2.7)	**		24.6 (2.6)	***^,#^		13.57	< 0.001
Religious (max score = 35)	14.6 (2.1)	16.8 (2.2)			16.6 (2.2)			1.86	NS
Nature (max score = 35)	26.7 (1.0)	27.9 (1.1)			28.4 (1.1)			2.15	NS
Creative (max score = 35)	16.5 (2.2)	15.8 (2.3)			18.2 (2.3)			3.32	NS
Biosocial (max score = 42)	29.1 (1.9)	29.3 (2.1)			28.1 (2.3)			0.96	NS
Total (max score = 182)	101 (4.6)	111 (5.4)	*		116 (6.1)	**		7.91	< 0.01
NEO Five-Factor Inventory (NEO-FFI)^c^								*F*_2*,*28_	*P=*
Neuroticism (max score = 48)	14.4 (2.0)	14.6 (1.5)			13.3 (1.5)			0.41	NS
Extraversion (max score = 48)	30.2 (1.4)	30.5 (2.0)			30. (2.0)			0.20	NS
Openness (max score = 48)	33.4 (2.2)	33.7 (2.3)			33.0 (2.5)			0.16	NS
Agreeableness (max score = 48)	33.1 (1.5)	32.6 (1.7)			33.8 (1.6)			1.09	NS
Conscientiousness (max score = 48)	30.1 (2.2)	31.8 (2.1)			34.8 (2.3)	*		4.48	< 0.05
State-Trait Anxiety Inventory (STAI)^c^								*F*_2*,*28_	*P=*
Trait score (max score = 80)	33.1 (2.3)	31.0 (1.8)			32.4 (1.8)			1.13	NS

*NS* not significant

^a^Data are expressed as percentage of maximum possible score. *N* = 14 subjects

^b^ *N* = 14 subjects

^c^ *N* = 15 subjects

*P < 0.05; **P < 0.01; ***P < 0.001, post hoc comparison with screening; ^#^ *P* < 0.05 compared with 1 month

#### Mysticism Scale, lifetime version

The MS total score and introvertive and extrovertive mysticism factor scores significantly increased at 1 and 12 months compared with pre-LSD screening (Table 1). No sex differences were observed.

#### Death Transcendence Scale

The DTS total score and mysticism subscale score significantly increased at 1 and 12 months compared with pre-LSD screening (Table 1). Mysticism ratings significantly increased after 12 months compared with 1 month. No sex differences were observed.

#### Personality measures

On the NEO-FFI, ratings of conscientiousness significantly increased at 12 months compared with pre-LSD screening (Table 1). No sex differences were observed. No differences in ratings of the other NEO-FFI factors were observed over time. On the STAI, no changes in trait scores were observed over time (Table 1).

#### Correlations between acute and long-term effects of LSD

Ratings of the acute effects of LSD on the MS, MEQ, and particularly 5D-ASC total score were positively associated with several long-term changes that were subjectively attributed to the LSD experience, which were assessed with the PEQ, MS, and DTS 12 months after LSD administration (Table 2). The overall acute mind-altering effects of LSD, reflected by the 5D-ASC total score, correlated with ratings of positive changes in mood (*R*_*s*_ = 0.56, *P* < 0.05), behavior (*R*_*s*_ = 0.53, *P* < 0.05), and well-being/life satisfaction (*R*_*s*_ = 0.53, *P* < 0.05) on the PEQ and changes in the MS total score (*R*_*s*_ = 0.62, *P* < 0.05) and DTS total score (*R*_*s*_ = 0.76, *P* < 0.01) at 12 months. Associations between the acute effects of LSD on ratings on the subscales of the 5D-ASC scale and the long-term effects of LSD are shown in Supplementary Table S2 and were consistent with those using the total 5D-ASC score.

**Table 2:** Correlations between the acute and long term effects of LSD

	Acute LSD effect		
	total MS score	total MEQ30 score	total 5D-ASC score
Long term LSD effect			
Persisting effects questionnaire (PE)			
Positive attitudes about life and/or self	0.30	0.50	0.48
Positive mood changes	0.33	0.51	**0.56**
Altruistic/positive social effects	0.04	0.25	0.27
Positive behavior changes	0.45	0.25	**0.53**
Personal meaning of experience	0.09	0.22	0.48
Spiritually significance of experience	0.41	0.29	0.52
Change in well-being/life satisfaction	0.51	**0.60**	**0.53**
Mysticism Scale (MS), lifetime			
Change in total MS score	0.38	0.36	**0.62**
Death Transcendence Scale (DTS)			
Change in total DTS score	***0.66***	**0.65**	***0.76***

Values are Spearman rank coefficients. Significant correlations are marked in bold (*P* < 0.05) or bold and italic (*P* < 0.01) font

*MS* Mysticism Scale, lifetime version, *MEQ30* 30-item Mystical Experience Questionnaire, *5D-ASC* Five Dimensions of Altered States of Consciousness Scale

Acute mystical-type effects, reflected by the MEQ30 total score, correlated with PEQ well-being/life satisfaction ratings at 12 months (*R*_*s*_ = 0.60, *P* < 0.05). The extent of acute mystical-type experiences, reflected by the MS total score and MEQ30 total score, also correlated with changes in DTS total scores at 12 months (*R*_*s*_ = 0.66, *P* < 0.01, and *R*_*s*_ = 0.65, *P* < 0.05, respectively).

#### Transient and lasting adverse effects

At the 1-month follow-up, one subject reported problems falling asleep and having more vivid dreams over 10 days after LSD administration. None of the participants reported any psychological problems or perceptual changes/disorders (e.g., flashbacks) up to 1 month after the LSD session. There were no spontaneous reports of adverse effects at the 12-month follow-up.

## Discussion

The main findings of the present study were that the administration of a single dose of LSD (200 μg p.o.) in healthy volunteers induced a subjectively meaningful experience with lasting positive effects that were attributed to the LSD experience by the participants. Greater ratings of acute LSD-induced alterations of mind on the 5D-ASC and/or mystical-type experiences on the MEQ30 scales were associated with greater ratings of well-being 12 months after the experience and changes in lifetime mystical experiences. LSD did not increase trait openness or produce relevant changes in personality measures. In the present study, LSD was not associated with lasting negative effects, as no lasting increases of negative attitudes, negative mood, and negative behavior could be observed after one and 12 months.

The present findings confirmed most of our hypotheses and complemented similar recent reports of lasting effects of psilocybin in healthy subjects using the same outcome measures (Griffiths et al. 2006, 2008, 2011, 2017; MacLean et al. 2011). The lasting effects of LSD were also reported in psychiatrically healthy subjects in older studies (McGlothlin et al. 1967). Short-term effects on personality measures were reported in one recent study (Carhart-Harris et al. 2016a). Specifically, in the older study by McGlothlin et al. (1967), psychological tests were administered before and 2 weeks and 6 months after three single dose sessions (200 μg LSD p.o. in each session) in 24 healthy subjects to explore potential changes in attitudes and values (McGlothlin et al. 1967). The participants were graduate students, were paid for participation, were LSD-naive, and received the drug in a secure setting but without suggestions of possible lasting effects (McGlothlin et al. 1967). In contrast to our study, the drug sessions were held in a “tastefully decorated room specifically designed to enhance the drug experience” without distractions by experiments (McGlothlin et al. 1967). A higher proportion of participants reported lasting effects on personality and greater appreciation of music and art 6 months after LSD administration compared with controls who received either amphetamine (20 mg p.o.) or a very low dose of LSD (25 μg p.o.; McGlothlin et al. 1967). The majority of the participants also rated the acute LSD response as a very dramatic and interesting experience. However, the pre-LSD vs. post-LSD comparison of a series of personality and creativity test ratings did not document relevant changes (McGlothlin et al. 1967). These previous findings of LSD-attributed subjective changes in attitudes, values, and esthetic interests in the absence of alterations in more objective test measures (McGlothlin et al. 1967) were largely confirmed by the present results.

More recently, Griffiths and colleagues administered a single dose of psilocybin in a supportive setting in 30 hallucinogen-naive and spiritually active healthy subjects to evaluate the long-term effects of psilocybin (Griffiths et al. 2006, 2008). A cross-over study design was used, including a control condition (methylphenidate, 40 mg/70 kg p.o.), to assess the acute effects of psilocybin (30 mg/70 kg p.o.). The MS and MEQ were used to assess acute mystical-type experiences (Barrett et al. 2015; Griffiths et al. 2006), similar to the present study (Liechti et al. 2017). Lasting effects of psilocybin were assessed at 2 and 14 months using the PEQ and MS (Griffiths et al. 2008). In contrast to the present study with LSD, the volunteers did not receive monetary compensation for participation. The investigators met with the participants on four occasions (for a total of 8 h) before the psilocybin session to prepare them for the experience. In contrast to the present study, this presession preparation explicitly included the monitor’s expectation that the psilocybin session could increase personal awareness and insight (Griffiths et al. 2006) and thus could have lasting positive effects. Additionally, all of the subjects participated at least intermittently in religious or spiritual activities, in which 56% of the volunteers reporting daily engagement, and 39% indicated at least monthly activities (Griffiths et al. 2006, 2008). Griffiths and colleagues also conducted a dose-effect study that included the administration of four different single doses of psilocybin and placebo in 18 participants and assessed lasting effects at 1 and 14 months using the PEQ, MS, DTS, and NEO Personality Inventory (NEO-PI) (Griffiths et al. 2011), similar to the present study. Similar to LSD in the present study, a single dose of psilocybin (30 mg/70 kg) significantly increased ratings of acute mystical-type experiences on the MS and MEQ (Barrett et al. 2015; Griffiths et al. 2006, 2011). However, 17 of the overall 54 participants also reported strong or extreme fear sometimes during the session after administration of psilocybin at this dose (Griffiths et al. 2006, 2011). Consistent with the present findings, psilocybin also produced significant positive effects but no negative effects on the PE compared with the control condition, which lasted up to 1, 2, and 14 months after the sessions (Griffiths et al. 2006, 2008, 2011). Total scores on the MS lifetime version increased 2 months after a single dose of psilocybin (Griffiths et al. 2006, 2008) and at the 14-month follow-up (Griffiths et al. 2008, 2011), similar to the present findings, in which MS scores increased 1 and 12 months after LSD administration. In the present study, we also observed a lasting increase in scores on the DTS Mysticism subscale, indicating an increase in mystical experiences, which is consistent with increases in lifetime mystical experiences on the MS, but no effects on the other subscale. In contrast, psilocybin only slightly changed scores on the DTS, with a slight increase on the religious subscale but not mysticism subscale at 14 months compared with pre-psilocybin screening (Griffiths et al. 2011).

Overall, the present study found no lasting effects of LSD on various personality trait measures 1 or 12 months after LSD administration. We did not confirm our study hypothesis that LSD would increase trait openness on the NEO-FFI. In contrast to the lack of long-term effects of LSD on personality in the present study, NEO-PI Openness scores increased 2 weeks after administration of a lower dose of LSD (75 μg i.v.) in healthy subjects with mostly significant prior LSD use (Carhart-Harris et al. 2016a). These mid-term personality changes were likely transient. Consistent with the present LSD follow-up results, psilocybin did not alter personality trait ratings 2 or 14 months after a single dose of psilocybin compared with pre-psilocybin screening (Griffiths et al. 2006, 2008). Although increases in openness were noted 14 months after psilocybin administration in a pooled analysis of both studies (MacLean et al. 2011), another more recent study by the same group again found no effects of psilocybin on NEO-PI personality measures 6 months after psilocybin administration (Griffiths et al. 2017). Trait anxiety ratings on the STAI were unchanged after LSD administration compared with pre-LSD screening in the present study. In contrast, LSD reduced trait anxiety ratings in patients with anxiety that was associated with life-threatening diseases (Gasser et al. 2014, 2015).

Altogether, the findings of controlled clinical studies, including the present study (Griffiths et al. 2006, 2008, 2011, 2017; MacLean et al. 2011; McGlothlin et al. 1967), are consistent with the view that serotonergic hallucinogens mainly produce lasting increases in lifetime mystical experiences and enduring positive effects on attitudes, mood, and behavior that are subjectively attributed to the hallucinogen experience. In contrast, the subjectively perceived changes did not result in relevant long-lasting changes in personality trait measures in healthy subjects.

The use of LSD and psilocybin has been associated with mystical experiences (Barrett and Griffiths 2017; Lyvers and Meester 2012). Mystical experiences that are induced by the hallucinogen psilocybin have been shown to be associated with long-term positive effects in healthy subjects (Griffiths et al. 2017; MacLean et al. 2011) and therapeutic outcomes in patients (Garcia-Romeu et al. 2015; Griffiths et al. 2016; Ross et al. 2016). An interesting line of investigation is to explore the factors that contribute to these mystical experiences and whether they specifically predict the long-term effects of hallucinogens. Mystical-type experiences predicted positive therapeutic outcomes in patients even after controlling for subjective intensity of the drug effect (Griffiths et al. 2016; Ross et al. 2016). Similar to previous studies with psilocybin in healthy subjects (Griffiths et al. 2008, 2017), the present study found that the long-term effects of LSD were associated with the extent of the acute response to LSD. However, the overall alterations of mind, reflected by 5D-ASC scores, better predicted the long-term effects of LSD compared with assessments of the more specific acute mystical-type experience, such as MS acute total scores or MEQ30 scores. Thus, the present findings indicate that the overall alterations in consciousness that are acutely induced by LSD may contribute to LSD’s lasting positive effects in normal subjects and in patients (Gasser et al. 2015; Liechti et al. 2017).

The extent of acute hallucinogen-induced mystical-type experiences has been shown to be mainly dose-dependent (Griffiths et al. 2011). Higher rates of meditation/spiritual practice or greater support for spiritual practice also increased ratings of acute mystical-type effects and contributed to the positive long-term effects compared with a group that received psilocybin but less spiritual support (Griffiths et al. 2017). In this study, spiritual practice suggestions to all participants included 10–30 min of daily meditation, awareness practice, journaling, and other activities personally judged to facilitate spiritual growth (Griffiths et al. 2017). However, high support for spiritual practice included dialog group sessions to discuss implementing and sustaining spiritual practices of meditation and spiritual awareness and a total of 35 h of guide-participant contact from study start to the 6-month follow-up compared with no group sessions and only 7 h of contact in the group with less spiritual support (Griffiths et al. 2017). Absolute ratings of the acute mystical-type effects of the hallucinogen on the MS and MEQ were generally higher in the studies by Griffiths and colleagues (Barrett et al. 2015; Griffiths et al. 2006, 2008, 2011, 2017) compared with the present study (Liechti et al. 2017). Importantly, this was the case in both the hallucinogen and control conditions, whereas the acute hallucinogen-induced increases in MS and MEQ scores relative to the control condition were greater after LSD than psilocybin administration (Barrett et al. 2015; Griffiths et al. 2006, 2011; Liechti et al. 2017). In the present study, LSD produced mean MEQ30 ratings of 61% and a complete mystical experience in only two subjects (12.5%; Liechti et al. 2017). In contrast, psilocybin produced mean MEQ30 score ratings of 77% and a complete mystical experience in 67% of the subjects (Barrett et al. 2015). However, placebo or active placebo (i.e., methylphenidate) also produced MEQ30 mean ratings of 23 and 33%, respectively, in these studies (Barrett et al. 2015), indicating relevant differences between the studies, including research subjects characteristics (set) and setting (Barrett and Griffiths 2017). In contrast to the studies by Griffiths and colleagues (Griffiths et al. 2006, 2011), the participants in the present study were mostly university students. They received monetary compensation for participation and were not required to be spiritually active (Liechti et al. 2017; Schmid et al. 2015). The participants in the present study had a personal or scientific interest in experiencing the mind-altering effects of the hallucinogen in a safe hospital environment, but no explicit expectations or suggestions of mystical or lasting effects were conveyed by the research team. Thus, these differences in the study populations (spiritually active vs. not explicitly active), preparation (suggestion of mystical/lasting effects vs. no suggestions), and setting (esthetic living room-like environment designed specifically for the study vs. hospital ward) likely accounted for the overall lower ratings of mystical-type effects in the present study compared with the studies by Griffiths and colleagues (Griffiths et al. 2006, 2011). We also observed no cases of relevant mystical-type effects after placebo administration (Liechti et al. 2017), whereas approximately 5% of the subjects in the studies by Griffiths and colleagues experienced a marked or total mystical experience after placebo (Barrett et al. 2015; Griffiths et al. 2011). Thus, the settings of the studies by Griffiths and colleagues appeared to be highly optimized to foster the emergence of mystical experiences (Barrett and Griffiths 2017), whereas fewer mystical-type experiences were reported in studies that were conducted by other groups (Carhart-Harris et al. 2016a; Gasser et al. 2015; Liechti et al. 2017; Studerus et al. 2011, 2012). Notably, the effects of LSD (200 μg) in the present study and psilocybin (30 mg/70 kg) on 5D-ASC scores were comparable (Griffiths et al. 2017; Liechti et al. 2017), indicating similar alterations in consciousness despite the different settings and acute mystical experiences. Remaining to be tested are what the high acute mystical experiences and associated long-term effects truly reflect. High MEQ scores (also after placebo) were observed in settings that involved more spiritual practice/support, and these scores and associated long-term changes may partly reflect such settings (Griffiths et al. 2017) and not the effects of psilocybin per se. Finally, the 5D-ASC and MEQ acute effect ratings were intercorrelated in the present study (Liechti et al. 2017). In fact, the acute effect ratings on both the 5D-ASC and MEQ significantly predicted long-term changes in subjective well-being and life satisfaction on the PEQ at 12 months in the present study. In contrast, MEQ ratings and thus specifically the mystical-type experiences appeared to mainly predict various long-term effects of psilocybin in more spiritually active subjects (Griffiths et al. 2008, 2017).

The present study has several limitations. First, the study did not include a true control condition for the long-term effects of LSD (i.e., a parallel control group for the long-term effects and not only for the acute effects of LSD). The lasting effects were subjectively attributed to the LSD experience and/or compared with measures prior to the LSD session over time and within-subjects. Thus, we cannot exclude the possibility that all of these subjective effects were attributable to expectations of positive long-term effects of LSD. Second, the study sample was small and not sufficiently powered to detect small effects on personality. Third, the questionnaires were previously used in English but were mostly not validated and the translations not validated in German. Forth, the study was conducted in healthy subjects and used many safety precautions (Johnson et al. 2008). Therefore, the results cannot necessarily be generalized to other settings or patients. Negative acute responses or negative long-term effects could occur in different populations or environments (Carbonaro et al. 2016; Halpern et al. 2016).

In conclusion, after 1 year, a single LSD experience produced personal meaning and enhanced well-being, which were subjectively attributed to the LSD experience, but no relevant changes in measures of personality traits.

## Electronic supplementary material

ESM 1 (PDF 51 kb)

ESM 2 (PDF 12 kb)

ESM 3 (PDF 17 kb)

Supplementary Table S1 (XLSX 18 kb)

Supplementary Table S2 (XLSX 13 kb)
