# Supplementary material for: Long-lasting subjective effects of LSD in normal subjects
Source: Psychopharmacology (Berl). 2017 Sep 16;235(2):535–45. doi: 10.1007/s00213-017-4733-3 (PMC5813062; doi:10.1007/s00213-017-4733-3)
Supplement: Supplementary file 1 — (PDF 51 kb) [file 213_2017_4733_MOESM1_ESM.pdf]

Persisting Effects Questionnaire

Das Ziel dieses Fragebogens ist es, anhaltende Auswirkungen zu erfassen, welche Sie auf die **Erfahrung mit der Substanz während Ihres Testtages sowie die anschliessende Reflexion derselbigen** zurückführen. Bitte geben Sie Ihre Einstellung **zum jetzigen Zeitpunkt** an.

Bitte beantworten Sie die Aussagen, indem Sie die folgende Beurteilungsskala benutzen. Umkreisen Sie die für Sie zutreffenden Antworten. Versuchen Sie, falls möglich, die Antwort "ich weiss nicht" zu vermeiden. Bitte beantworten Sie alle Fragen.

0 - gar nicht, überhaupt nicht
1 - so wenig, dass ich mich nicht entscheiden kann
2 - wenig
3 - mässig
4 - stark und mehr als jegliche früheren Erfahrungen
5 - extrem, mehr als für menschenmöglich gehalten

Deutsche Übersetzung des Persisting Effects Questionnaire (R. Griffiths) durch Schmid und Liechti

1	Die Erfahrung hat meine Lebensphilosophie positiv beeinflusst.	**0**	**1**	**2**	**3**	**4**	**5**
2	Die Erfahrung hat meine Lebensphilosophie negativ beeinflusst.	**0**	**1**	**2**	**3**	**4**	**5**
3	Meine Wertschätzung des Lebens hat zugenommen.	**0**	**1**	**2**	**3**	**4**	**5**
4	Meine Wertschätzung des Lebens hat abgenommen.	**0**	**1**	**2**	**3**	**4**	**5**
5	Mein Leben hat an dynamischer Qualität abgenommen.	**0**	**1**	**2**	**3**	**4**	**5**
6	Mein Leben hat an dynamischer Qualität zugenommen.	**0**	**1**	**2**	**3**	**4**	**5**
7	Mein Leben hat an Bedeutung verloren.	**0**	**1**	**2**	**3**	**4**	**5**
8	Mein Leben hat an Bedeutung gewonnen.	**0**	**1**	**2**	**3**	**4**	**5**
9	Ich habe weniger Enthusiasmus für das Leben im Allgemeinen.	**0**	**1**	**2**	**3**	**4**	**5**
10	Ich habe mehr Enthusiasmus für das Leben im Allgemeinen.	**0**	**1**	**2**	**3**	**4**	**5**
11	Meine Wertschätzung hat sich positiv verändert (z.B. in Bezug darauf, was im Leben wichtig ist).	**0**	**1**	**2**	**3**	**4**	**5**
12	Meine Wertschätzung hat sich negativ verändert (z.B. in Bezug darauf, was im Leben wichtig ist).	**0**	**1**	**2**	**3**	**4**	**5**
13	Mein Leben hat an Reichtum zugenommen.	**0**	**1**	**2**	**3**	**4**	**5**
14	Mein Leben hat an Reichtum abgegeben.	**0**	**1**	**2**	**3**	**4**	**5**
15	Ich habe mehr Freude im Leben.	**0**	**1**	**2**	**3**	**4**	**5**
16	Ich habe weniger Freude im Leben.	**0**	**1**	**2**	**3**	**4**	**5**
17	Die Erfahrung war hinderlich für mein Leben.	**0**	**1**	**2**	**3**	**4**	**5**
18	Die Erfahrung war wertvoll für mein Leben.	**0**	**1**	**2**	**3**	**4**	**5**
19	Ich bin optimistischer.	**0**	**1**	**2**	**3**	**4**	**5**
20	Ich bin weniger optimistisch.	**0**	**1**	**2**	**3**	**4**	**5**
21	Ich habe mehr Vertrauen gewonnen, dass alles im Leben gut	**0**	**1**	**2**	**3**	**4**	**5**

	kommen wird.						
22	Ich habe Vertrauen darin verloren, dass alles im Leben gut kommen wird.	**0**	**1**	**2**	**3**	**4**	**5**
23	Ich bin mehr besorgt über meine Zukunft.	**0**	**1**	**2**	**3**	**4**	**5**
24	Ich bin weniger besorgt über meine Zukunft.	**0**	**1**	**2**	**3**	**4**	**5**
25	Ich mache mir weniger Sorgen über meine Vergangenheit.	**0**	**1**	**2**	**3**	**4**	**5**
26	Ich mache mir mehr Sorgen über meine Vergangenheit.	**0**	**1**	**2**	**3**	**4**	**5**
27	Ich fühle mehr persönliche Integrität.	**0**	**1**	**2**	**3**	**4**	**5**
28	Ich fühle weniger persönliche Integrität.	**0**	**1**	**2**	**3**	**4**	**5**
29	Ich habe ein vermindertes Bewusstsein innerer Autorität in meinem Leben.	**0**	**1**	**2**	**3**	**4**	**5**
30	Ich habe ein vermehrtes Bewusstsein innerer Autorität in meinem Leben.	**0**	**1**	**2**	**3**	**4**	**5**
31	Ich fühle mich weniger kreativ.	**0**	**1**	**2**	**3**	**4**	**5**
32	Ich fühle mich kreativer.	**0**	**1**	**2**	**3**	**4**	**5**
33	Ich bin authentischer geworden.	**0**	**1**	**2**	**3**	**4**	**5**
34	Ich bin weniger authentisch geworden.	**0**	**1**	**2**	**3**	**4**	**5**
35	Ich habe mehr gute Laune, Lebenslust und Leichtigkeit im Sein.	**0**	**1**	**2**	**3**	**4**	**5**
36	Ich habe weniger gute Laune, Lebenslust und Leichtigkeit im Sein.	**0**	**1**	**2**	**3**	**4**	**5**
37	Ich habe weniger Geduld und eine niedrigere Frustrationstoleranz.	**0**	**1**	**2**	**3**	**4**	**5**
38	Ich bin geduldiger und toleranter gegenüber Frustration geworden.	**0**	**1**	**2**	**3**	**4**	**5**
39	Ich bin mental weniger flexibel und offen.	**0**	**1**	**2**	**3**	**4**	**5**
40	Ich habe an mentaler Flexibilität und Offenheit gewonnen.	**0**	**1**	**2**	**3**	**4**	**5**
41	Ich bin selbstsicherer.	**0**	**1**	**2**	**3**	**4**	**5**
42	Ich bin weniger selbstsicher.	**0**	**1**	**2**	**3**	**4**	**5**
43	Ich bin ehrlicher zu mir selbst.	**0**	**1**	**2**	**3**	**4**	**5**
44	Ich bin weniger ehrlich zu mir selbst.	**0**	**1**	**2**	**3**	**4**	**5**
45	Ich bin weniger gut fähig, mein Leiden zu tolerieren.	**0**	**1**	**2**	**3**	**4**	**5**
46	Ich bin besser fähig, mein Leiden zu tolerieren.	**0**	**1**	**2**	**3**	**4**	**5**
47	Ich mache mir weniger Sorgen und Gedanken über meinen Körper.	**0**	**1**	**2**	**3**	**4**	**5**
48	Ich mache mir mehr Sorgen und Gedanken über meinen Körper.	**0**	**1**	**2**	**3**	**4**	**5**
49	Gefühle wie Traurigkeit oder Depression haben abgenommen.	**0**	**1**	**2**	**3**	**4**	**5**
50	Gefühle wie Traurigkeit oder Depression haben zugenommen.	**0**	**1**	**2**	**3**	**4**	**5**
51	Gefühle wie Angst oder Nervosität haben abgenommen.	**0**	**1**	**2**	**3**	**4**	**5**
52	Gefühle wie Angst oder Nervosität haben zugenommen.	**0**	**1**	**2**	**3**	**4**	**5**
53	Ich verspüre mehr Liebe und Offenherzigkeit.	**0**	**1**	**2**	**3**	**4**	**5**
54	Ich verspüre weniger Liebe und Offenherzigkeit.	**0**	**1**	**2**	**3**	**4**	**5**

55	Ich erlebe weniger inneren Frieden (innere Ruhe, Heiterkeit, Gelassenheit, Zufriedenheit).	**0**	**1**	**2**	**3**	**4**	**5**
56	Ich erlebe mehr inneren Frieden (innere Ruhe, Heiterkeit, Gelassenheit, Zufriedenheit).	**0**	**1**	**2**	**3**	**4**	**5**
57	Ich habe weniger positive Gefühle (z.B. Liebe Freude, Zufriedenheit, Dankbarkeit).	**0**	**1**	**2**	**3**	**4**	**5**
58	Ich habe mehr positive Gefühle (z.B. Liebe, Freude, Zufriedenheit, Dankbarkeit).	**0**	**1**	**2**	**3**	**4**	**5**
59	Ich bin inspirierter.	**0**	**1**	**2**	**3**	**4**	**5**
60	Ich bin weniger inspiriert.	**0**	**1**	**2**	**3**	**4**	**5**
61	Ich empfinde mehr Gefühle von Ärger (z.B. Bitterkeit, Feindseligkeit, Zorn).	**0**	**1**	**2**	**3**	**4**	**5**
62	Ich empfinde weniger Gefühle von Ärger (z.B. Bitterkeit, Feindseligkeit, Zorn).	**0**	**1**	**2**	**3**	**4**	**5**
63	Ich habe vermehrt Schuldgefühle.	**0**	**1**	**2**	**3**	**4**	**5**
64	Ich habe weniger Schuldgefühle.	**0**	**1**	**2**	**3**	**4**	**5**
65	Ich empfinde mehr Freude.	**0**	**1**	**2**	**3**	**4**	**5**
66	Ich empfinde weniger Freude.	**0**	**1**	**2**	**3**	**4**	**5**
67	Ich bin sensibler geworden für die Bedürfnisse anderer.	**0**	**1**	**2**	**3**	**4**	**5**
68	Ich bin weniger sensibel für die Bedürfnisse anderer geworden.	**0**	**1**	**2**	**3**	**4**	**5**
69	Ich habe weniger das Bedürfnis anderen zu dienen.	**0**	**1**	**2**	**3**	**4**	**5**
70	Ich habe vermehrt das Bedürfnis anderen zu dienen.	**0**	**1**	**2**	**3**	**4**	**5**
71	Ich bin anderen gegenüber toleranter.	**0**	**1**	**2**	**3**	**4**	**5**
72	Ich bin anderen gegenüber weniger tolerant.	**0**	**1**	**2**	**3**	**4**	**5**
73	Meine Beziehung zu anderen ist insgesamt positiver.	**0**	**1**	**2**	**3**	**4**	**5**
74	Meine Beziehung zu anderen ist insgesamt negativer.	**0**	**1**	**2**	**3**	**4**	**5**
75	Ich zeige mehr Feindseligkeit anderen gegenüber.	**0**	**1**	**2**	**3**	**4**	**5**
76	Ich zeige anderen mehr Zuwendung.	**0**	**1**	**2**	**3**	**4**	**5**
77	Meine Wahrnehmung zwischenmenschlicher Beziehungen hat abgenommen (z.B. Empathie, Sensibilität).	**0**	**1**	**2**	**3**	**4**	**5**
78	Meine Wahrnehmung zwischenmenschlicher Beziehungen hat zugenommen (z.B. Empathie, Sensibilität).	**0**	**1**	**2**	**3**	**4**	**5**
79	Meine negative Äusserung von Ärger hat abgenommen (z. B. sich über andere lustig machen, Reizbarkeit gegenüber anderen zeigen).	**0**	**1**	**2**	**3**	**4**	**5**
80	Meine negative Äusserung von Ärger hat zugenommen (z. B. sich über andere lustig machen, Reizbarkeit gegenüber anderen zeigen).	**0**	**1**	**2**	**3**	**4**	**5**
81	Mein soziales Mitgefühl hat abgenommen.	**0**	**1**	**2**	**3**	**4**	**5**
82	Mein soziales Mitgefühl hat zugenommen.	**0**	**1**	**2**	**3**	**4**	**5**
83	Meine Sorgen um verletzbare Menschen haben abgenommen.	**0**	**1**	**2**	**3**	**4**	**5**
84	Meine Sorgen um verletzbare Menschen haben zugenommen.	**0**	**1**	**2**	**3**	**4**	**5**
85	Mein Verhalten hat sich in einer Art verändert, welche ich als	**0**	**1**	**2**	**3**	**4**	**5**

	negativ beurteilen würde.						
86	Mein Verhalten hat sich in einer Art verändert, welche ich als positiv beurteilen würde.	0	1	2	3	4	5
87	Ich verspüre vermehrt Ehrfurcht.	0	1	2	3	4	5
88	Ich verspüre weniger Ehrfurcht.	0	1	2	3	4	5
89	Meine Würdigung der gesamten Schöpfung hat abgenommen.	0	1	2	3	4	5
90	Meine Würdigung der gesamten Schöpfung hat zugenommen.	0	1	2	3	4	5
91	Ich verbringe weniger Zeit in stiller Meditation oder mit Gebeten.	0	1	2	3	4	5
92	Ich verbringe mehr Zeit in stiller Meditation oder mit Gebeten.	0	1	2	3	4	5
93	Ich verspüre ein stärkeres Bewusstsein für die Kostbarkeit des Lebens.	0	1	2	3	4	5
94	Ich verspüre ein geringeres Bewusstsein für die Kostbarkeit des Lebens.	0	1	2	3	4	5
95	Ich verspüre seltener tiefe Heiligkeit.	0	1	2	3	4	5
96	Ich verspüre öfters tiefe Heiligkeit.	0	1	2	3	4	5
97	Ich glaube jetzt weniger an eine Form von Fortdauer nach dem Tod.	0	1	2	3	4	5
98	Ich glaube jetzt mehr an eine Form von Fortdauer nach dem Tod.	0	1	2	3	4	5
99	Ich bin weniger ängstlich bezüglich meines eigenen Todes.	0	1	2	3	4	5
100	Ich bin ängstlicher bezüglich meines eigenen Todes.	0	1	2	3	4	5
101	Ich gebe meinem spirituellen / religiösen Verständnis weniger Gewicht.	0	1	2	3	4	5
102	Ich gebe meinem spirituellen / religiösen Verständnis mehr Gewicht.	0	1	2	3	4	5
103	Meine Erfahrung von Heiligkeit im alltäglichen Leben hat abgenommen.	0	1	2	3	4	5
104	Meine Erfahrung von Heiligkeit im alltäglichen Leben hat zugenommen.	0	1	2	3	4	5
105	Ich empfinde eine tiefere Dankbarkeit für das Leben.	0	1	2	3	4	5
106	Ich empfinde weniger Dankbarkeit für das Leben.	0	1	2	3	4	5
107	Ich verspüre ein erhöhte Wahrnehmung einer persönlichen Beziehung zu Gott (höhere Macht) / ultimativen Realität.	0	1	2	3	4	5
108	Ich verpüre eine verminderte Wahrnehmung einer persönlichen Beziehung zu Gott (höhere Macht) / ultimativen Realität.	0	1	2	3	4	5
109	Ich empfinde eine verstärkte Verbindung zu Gott (höhere Macht).	0	1	2	3	4	5
110	Ich empfinde eine verminderte Verbindung zu Gott (höhere Macht).	0	1	2	3	4	5
111	Spiritualität ist in meinem Leben zentraler geworden.	0	1	2	3	4	5
112	Spiritualität ist in meinem Leben weniger zentral geworden.	0	1	2	3	4	5
113	Meine Gebete und / oder Meditationen geben mir weniger emotionalen Rückhalt.	0	1	2	3	4	5
114	Meine Gebete und / oder Meditationen geben mir mehr emotionalen Rückhalt.	0	1	2	3	4	5
115	Ich möchte mehr Distanz zu Gott (höhere Macht / Realität).	0	1	2	3	4	5

116	Ich möchte weniger Distanz zu Gott (höheren Macht / Realität).	**0**	**1**	**2**	**3**	**4**	**5**
117	Ich fühle mich im Universum mehr zu Hause.	**0**	**1**	**2**	**3**	**4**	**5**
118	Ich fühle mich weniger zu Hause im Universum.	**0**	**1**	**2**	**3**	**4**	**5**
119	Ich habe weniger das Gefühl, dass alles im Leben miteinander verbunden ist.	**0**	**1**	**2**	**3**	**4**	**5**
120	Ich habe vermehrt das Gefühl, dass alles im Leben miteinander verbunden ist.	**0**	**1**	**2**	**3**	**4**	**5**
121	Ich habe ein vermehrtes Gefühl einer universellen Ordnung, welche das menschliche Vorstellungsvermögen übersteigt.	**0**	**1**	**2**	**3**	**4**	**5**
122	Ich habe weniger das Gefühl, dass eine universelle Ordnung existiert, die das menschliche Vorstellungsvermögen übersteigt.	**0**	**1**	**2**	**3**	**4**	**5**
123	Ich habe einen vermehrten Glauben an eine höhere Ebene des Bewusstseins oder der Spiritualität, welche alle Menschen verbindet.	**0**	**1**	**2**	**3**	**4**	**5**
124	Ich habe einen verminderten Glauben an eine höhere Ebene des Bewusstseins oder der Spiritualität, welche alle Menschen verbindet.	**0**	**1**	**2**	**3**	**4**	**5**
125	Ich habe ein vermindertes Gefühl einer emotionalen Verbundenheit mit der gesamten Menschheit.	**0**	**1**	**2**	**3**	**4**	**5**
126	Ich habe ein verstärktes Gefühl einer emotionalen Verbundenheit mit der gesamten Menschheit.	**0**	**1**	**2**	**3**	**4**	**5**
127	Ich fühle mich mehr angekommen im Hier und Jetzt.	**0**	**1**	**2**	**3**	**4**	**5**
128	Ich fühle mich weniger angekommen im Hier und Jetzt.	**0**	**1**	**2**	**3**	**4**	**5**
129	Ich erlebe eine erhöhte emotionale Nähe zu jemandem der bereits verstorben ist.	**0**	**1**	**2**	**3**	**4**	**5**
130	Ich verbringe weniger Zeit mit andächtigen Tätigkeiten.	**0**	**1**	**2**	**3**	**4**	**5**
131	Ich verbringe mehr Zeit mit andächtigen Tätigkeiten.	**0**	**1**	**2**	**3**	**4**	**5**
132	Ich verspüre ein vermehrtes berufliches Pflichtgefühl.	**0**	**1**	**2**	**3**	**4**	**5**
133	Ich verspüre ein vermindertes berufliches Pflichtgefühl.	**0**	**1**	**2**	**3**	**4**	**5**
134	Ich lege vermehrt Wert auf traditionelle theologische Erklärungen um spirituelle Einsichten auszudrücken.	**0**	**1**	**2**	**3**	**4**	**5**
135	Ich lege weniger Wert auf traditionelle theologische Erklärungen um spirituelle Einsichten auszudrücken.	**0**	**1**	**2**	**3**	**4**	**5**
136	Ich habe durch die Erfahrung etwas Nützliches gelernt.	**0**	**1**	**2**	**3**	**4**	**5**
137	Ich habe das Gefühl, eine neue Dimension des Lebens kennengelernt zu haben.	**0**	**1**	**2**	**3**	**4**	**5**
138	Rückblickend bin ich überzeugt, das ich mit der ultimativen Realität in Kontakt kam (z.B. ich habe "gesehen" / "gewusst" was die ultimative Realität ist)	**0**	**1**	**2**	**3**	**4**	**5**
139	Ich habe nun das Gefühl, dass die Bedeutung meiner Erfahrung jenseits dessen liegt, was mit Worten beschrieben werden kann.	**0**	**1**	**2**	**3**	**4**	**5**
140	Ich habe nun das Gefühl, dass mein Leben aufgrund der Erfahrung einen deutlichen Richtungswechsel eingeschlagen hat.	**0**	**1**	**2**	**3**	**4**	**5**

Bitte umkreisen Sie die für Sie zutreffende Antwort in den folgenden abschliessenden Fragen:

1. Wie bedeutungsvoll war das Erlebnis für Sie persönlich?
    a. Nicht mehr als Routine, alltägliche Erfahrungen
    b. Ähnlich wichtig wie Erfahrungen, die bei mir ungefähr wöchentlich oder mehrmals wöchentlich vorkommen
    c. Ähnlich wichtig wie Erfahrungen, die mir durchschnittlich monatlich passieren.
    d. Ähnlich wichtig wie Erfahrungen, wie sie ungefähr einmal pro Jahr vorkommen.
    e. Ähnlich wichtig wie Erfahrungen, die ungefähr alle 5 Jahre vorkommen.
    f. Unter den 10 wichtigsten Erfahrungen meines Lebens.
    g. Unter den 5 wichtigsten Erfahrungen meines Lebens.
    h. Die wichtigste Erfahrung meines Lebens.

2. A. Geben Sie den Grad an, wie spirituell bedeutungsvoll die Erfahrung für Sie war:
    a. Überhaupt nicht
    b. Kaum
    c. Mittelmässig
    d. Sehr stark
    e. Unter den 5 spirituell bedeutungsvollsten Erfahrungen in Ihrem Leben
    f. Die spirituellste Erfahrung in Ihrem Leben

   B. Falls die Erfahrung spirituell wichtig war, geben Sie an in welchem Ausmass:
    g. Nicht einzigartig (ähnlich wie andere spirituelle Erfahrungen, die ich bereits hatte)
    h. Ungewöhnlich (nicht vergleichbar mit anderen spirituellen Erfahrungen)
    i. Komplett einzigartig (anders als alle meine bisherigen spirituellen Erfahrungen)

3. Haben Sie das Gefühl, dass die Erfahrung und diese Auseinandersetzung damit zu Veränderungen in Ihrem persönlichen Wohlbefinden oder Ihrer Zufriedenheit geführt haben?
    a. Stark zugenommen
    b. Mässig zugenommen
    c. Leicht zugenommen
    d. Keine Veränderung
    e. Leicht abgenommen
    f. Mässig abgenommen
    g. Stark abgenommen
