# Supplementary material for: Long-lasting subjective effects of LSD in normal subjects
Source: Psychopharmacology (Berl). 2017 Sep 16;235(2):535–45. doi: 10.1007/s00213-017-4733-3 (PMC5813062; doi:10.1007/s00213-017-4733-3)
Supplement: Supplementary file 2 — (PDF 12 kb) [file 213_2017_4733_MOESM2_ESM.pdf]

Mysticism Scale (life-time)

Bitte füllen Sie diesen Fragebogen hinsichtlich aller Erfahrungen, welche Sie bisher in Ihrem Leben gemacht haben, aus.

Beantworten Sie wie stark die Beschreibung auf Ihre bisherigen Erlebnisse zutrifft. Kreisen Sie die Antwort auf der Skala von +4 bis -4 ein, entsprechend der unten aufgeführten Bewertungsskala. Markieren Sie alle Aussagen. Versuchen Sie, falls möglich, „ich weiss nicht“ zu vermeiden. Die Aussagen beziehen sich nicht nur auf eine einzige Erfahrung, sondern mögliche Erfahrungen, welche Sie **im Verlaufe ihres gesamten Lebens bisher** gemacht haben.

+4: extrem zutreffend
+3: sehr zutreffend
+2: mittelstark zutreffend
+1: leicht zutreffend
0: Ich weiss nicht
-1: leicht nicht zutreffend
-2: mittelstark nicht zutreffend
-3: sehr unzutreffend
-4: extrem unzutreffend

Deutsche Übersetzung der Hood Mysticism Scale durch Schmid und Liechti

	extrem zutreffend	sehr zutreffend	mittel-stark zutreffend	leicht zutreffend	Ich weiss nicht	leicht nicht zutreffend	mittel-stark nicht zutreffend	sehr unzu-treffend	extrem unzu-treffend
1. Mein Erlebnis war zeitlos und raumlos.	+4	+3	+2	+1	0	-1	-2	-3	-4
2. Ich hatte NIE eine Erfahrung, welche ich nicht mit Worten beschreiben könnte.	+4	+3	+2	+1	0	-1	-2	-3	-4
3. Ich erlebte, wie etwas Grösseres als ich selbst mich aufnahm.	+4	+3	+2	+1	0	-1	-2	-3	-4
4. Ich erlebte, wie alles aus meinem Bewusstsein zu verschwinden schien, bis ich nur noch der Leere bewusst war.	+4	+3	+2	+1	0	-1	-2	-3	-4
5. Ich habe tiefe Freude empfunden.	+4	+3	+2	+1	0	-1	-2	-3	-4
6. Ich hatte NIE ein Erlebnis, bei dem ich das Gefühl hatte mit allem Eins zu sein.	+4	+3	+2	+1	0	-1	-2	-3	-4
7. Ich habe NIE einen Zustand tiefsten Friedens erlebt.	+4	+3	+2	+1	0	-1	-2	-3	-4
8. Ich hatte NIE eine Erfahrung, in welcher ich das Gefühl hatte, dass alle Dinge lebendig wären.	+4	+3	+2	+1	0	-1	-2	-3	-4
9. Ich hatte NIE ein Erlebnis, welches mir heilig erschien.	+4	+3	+2	+1	0	-1	-2	-3	-4
10. Ich hatte NIE das Gefühl, dass die Dinge ein Bewusstsein hätten.	+4	+3	+2	+1	0	-1	-2	-3	-4

	extrem zutreffend	sehr zutreffend	mittel-stark zutreffend	leicht zutreffend	Ich weiss nicht	leicht nicht zutreffend	mittel-stark nicht zutreffend	sehr unzu-treffend	extrem unzu-treffend
11. Ich erlebte, jeglichen Sinn für Zeit oder Raum verloren zu haben.	+4	+3	+2	+1	0	-1	-2	-3	-4
12. Ich hatte eine Erfahrung, wo ich realisierte mit allen Dingen Eins zu sein.	+4	+3	+2	+1	0	-1	-2	-3	-4
13. Ich hatte ein Erlebnis, in welchem sich mir eine neue Sicht der Realität offenbarte.	+4	+3	+2	+1	0	-1	-2	-3	-4
14. Ich habe NIE etwas als Göttlich erlebt.	+4	+3	+2	+1	0	-1	-2	-3	-4
15. Ich hatte NIE ein Erlebnis, in welchem Zeit und Raum nicht existierten.	+4	+3	+2	+1	0	-1	-2	-3	-4
16. Ich hatte NIE eine Erfahrung, welche ich als endgültige Realität bezeichnen könnte.	+4	+3	+2	+1	0	-1	-2	-3	-4
17. Ich hatte eine Erfahrung, in welcher mir die endgültige Realität offenbart wurde.	+4	+3	+2	+1	0	-1	-2	-3	-4
18. Ich hatte eine Erfahrung wo für einen Moment alles vollkommen war.	+4	+3	+2	+1	0	-1	-2	-3	-4
19. Ich hatte ein Gefühl, alles in der Welt sei Teil eines gemeinsamen Ganzen.	+4	+3	+2	+1	0	-1	-2	-3	-4
20. Ich hatte ein Erlebnis wo ich wusste, dass es heilig ist.	+4	+3	+2	+1	0	-1	-2	-3	-4
21. Ich hatte NIE eine Erfahrung, welche ich nicht mit Worten beschreiben konnte.	+4	+3	+2	+1	0	-1	-2	-3	-4
22. Ich hatte ein Erlebnis, welches mich mit einem Gefühl der Ehrfurcht hinterliess.	+4	+3	+2	+1	0	-1	-2	-3	-4
23. Ich hatte ein Erlebnis, welches unmöglich beschrieben werden kann.	+4	+3	+2	+1	0	-1	-2	-3	-4
24. Ich hatte NIE das Gefühl, dass sich mein eigenes Selbst mit etwas Grösserem zusammenschloss.	+4	+3	+2	+1	0	-1	-2	-3	-4
25. Ich hatte NIE ein Erlebnis, welches mich in einem Gefühl des Staunens zurückliess.	+4	+3	+2	+1	0	-1	-2	-3	-4
26. Ich hatte NIE eine Erfahrung, wo sich mir tiefere Ansichten der Realität zeigten.	+4	+3	+2	+1	0	-1	-2	-3	-4

	extrem extrem zutreffend	sehr sehr zutreffend	mittel- mittel- stark zutreffend	leicht leicht zutreffend	Ich Ich weiss nicht	leicht leicht nicht zutreffend	mittel- mittel- stark nicht zutreffend	sehr sehr unzu- treffend	extrem extrem unzu- treffend
27. Ich hatte NIE ein Erlebnis, in welchem Zeit, Raum und Distanz bedeutungslos erschienen.	+4	+3	+2	+1	0	-1	-2	-3	-4
28. Ich hatte NIE ein Erlebnis, in welchem ich der Einheit aller Dinge bewusst wurde.	+4	+3	+2	+1	0	-1	-2	-3	-4
29. Ich hatte eine Erfahrung, in welcher alle Dinge ein Bewusstsein zu besitzen schienen.	+4	+3	+2	+1	0	-1	-2	-3	-4
30. Ich hatte NIE eine Erfahrung, während welcher alle Dinge sich zu einem grossen Ganzen zu vereinen schienen.	+4	+3	+2	+1	0	-1	-2	-3	-4
31. Ich hatte das Gefühl, dass nichts jemals wirklich tot ist.	+4	+3	+2	+1	0	-1	-2	-3	-4
32. Ich hatte eine Erfahrung, welche nicht in Worten ausgedrückt werden kann.	+4	+3	+2	+1	0	-1	-2	-3	-4
