# Supplementary material for: Long-lasting subjective effects of LSD in normal subjects
Source: Psychopharmacology (Berl). 2017 Sep 16;235(2):535–45. doi: 10.1007/s00213-017-4733-3 (PMC5813062; doi:10.1007/s00213-017-4733-3)
Supplement: Supplementary file 3 — (PDF 17 kb) [file 213_2017_4733_MOESM3_ESM.pdf]

Death Transcendence Scale

Bitte füllen Sie diesen Fragebogen hinsichtlich aller Erfahrungen, welche Sie bisher in Ihrem Leben gemacht haben, aus.

Bitte beantworten Sie die Aussagen, indem Sie die folgende Beurteilungsskala benutzen. Umkreisen Sie die für Sie zutreffenden Antworten. Versuchen Sie, falls möglich, die Antwort "ich weiss nicht" zu vermeiden.

+3:	stimme stark zu
+2:	stimme zu
+1:	stimme eher zu
0:	Ich weiss nicht
-1:	stimme eher nicht zu
-2:	stimme nicht zu
-3:	stimme überhaupt nicht zu

Deutsche Übersetzung der Death Transcendence Scale (VandeCreek) durch Schmid und Liechti

	Stimme stark zu	stimme zu	stimme eher zu	ich weiss nicht	stimme eher nicht zu	stimme nicht zu	stimme überhaupt nicht zu
1. Wenn ich nie etwas Bedeutsames mache, wird mein Leben verschwendet sein.	+3	+2	+1	0	-1	-2	-3
2. Ohne Kinder ist das Leben unvollständig.	+3	+2	+1	0	-1	-2	-3
3. Ich hatte ein Erlebnis, in welchem ich fühlte, dass alles in der Welt Teil eines gemeinsamen Ganzen ist.	+3	+2	+1	0	-1	-2	-3
4. Ich hatte nie ein Erlebnis, in welchem sich alle Dinge zu einem Ganzen zu vereinen schienen.	+3	+2	+1	0	-1	-2	-3
5. Mein Tod beendet meine persönliche Existenz nicht.	+3	+2	+1	0	-1	-2	-3
6. Bäche, Bäume und Menschen sind alle Eins in der Natur.	+3	+2	+1	0	-1	-2	-3
7. Es gibt eine Kraft oder Macht, welche sowohl das Leben als auch den Tod kontrolliert und ihnen Bedeutung verleiht.	+3	+2	+1	0	-1	-2	-3
8. Ich hatte nie ein Erlebnis, in welchem ich mir der Einheit aller Dinge bewusst wurde.	+3	+2	+1	0	-1	-2	-3

	Stimme stark zu	stimme zu	stimme eher zu	ich weiss nicht	stimme eher nicht zu	stimme nicht zu	stimme überhaupt nicht zu
9. Nur die Natur ist für ewig.	+3	+2	+1	0	-1	-2	-3
10. Wenn andere, die ich liebe, sich nach meinem Tod nicht mehr an mich erinnern, war mein Leben verschwendet.	+3	+2	+1	0	-1	-2	-3
11. Ich hatte ein Erlebnis, in welchem ich die Einheit von mir mit allen Dingen erkannte.	+3	+2	+1	0	-1	-2	-3
12. Tod ist nie nur ein Ende, sondern ein Teil eines Prozesses.	+3	+2	+1	0	-1	-2	-3
13. Nach meinem Tod wird vieles von mir durch. meine Kinder weiterleben.	+3	+2	+1	0	-1	-2	-3
1. Ich mag sterben, aber die Flüsse und Berge bleiben.	+3	+2	+1	0	-1	-2	-3
15. Ich glaube an ein Leben nach dem Tod.	+3	+2	+1	0	-1	-2	-3
16. Bedeutungslose Arbeit führt zu einem bedeutungslosen Leben.	+3	+2	+1	0	-1	-2	-3
17. Ich hatte nie ein Erlebnis, in welchem ich mich mit allen Dingen verbunden und Eins fühlte.	+3	+2	+1	0	-1	-2	-3
18. Kreativ sein heisst für immer leben.	+3	+2	+1	0	-1	-2	-3
19. Der Tod ist so natürlich wie alles Andere in der Natur.	+3	+2	+1	0	-1	-2	-3
20. Mein Leben mag enden, aber was wichtig ist wird durch meine Familie weiterleben.	+3	+2	+1	0	-1	-2	-3
21. Ohne Kinder wäre vieles, was im Leben am wertvollsten ist verschwendet.	+3	+2	+1	0	-1	-2	-3
22. Feste Beziehungen mit Familie und Freunden ist ein beständiger Wert.	+3	+2	+1	0	-1	-2	-3
23. Beziehungen mit Familie und Freunden sind unter den beständigsten Werten.	+3	+2	+1	0	-1	-2	-3

	Stimme stark zu	stimme zu	stimme eher zu	ich weiss nicht	stimme eher nicht zu	stimme nicht zu	stimme überhaupt nicht zu
24. Der Tod ist ein Übergang zu etwas noch Grösserem als dieses Leben.	+3	+2	+1	0	-1	-2	-3
25. Was auch immer kommt, wir sind alle Teil der Natur.	+3	+2	+1	0	-1	-2	-3
26. Für mich ist es wichtig, im Leben etwas zu tun, wofür man sich nach meinem Tod an mich erinnert.	+3	+2	+1	0	-1	-2	-3
